# The preponderance of major depressive disorder among women of reproductive age and the clinical utility of sertraline

**DOI:** 10.1192/j.eurpsy.2024.1111

**Published:** 2024-08-27

**Authors:** A. Fagiolini, M. P. Mariano, E. Biesheuvel, P. Purushottamahanti

**Affiliations:** ^1^Department of Molecular Medicine, University of Siena School of Medicine, Siena, Italy; ^2^Department of Psychiatry, University of the East Ramon Magsaysay Memorial Medical Center, Quezon City, Philippines; ^3^Global Clinical Operations, Viatris Inc, Amstelveen, Netherlands; ^4^Global Medical Affairs, Viatris Inc, Bangalore, India

## Abstract

**Introduction:**

Major depressive disorder (MDD) is twice as common in women than men and is more frequently reported during their reproductive years (Shi *et al.* Front. Psychiatr 2021; 12 589687).  MDD affects up to 12.7% of pregnant women and can significantly impact foetal and maternal health. Hence, clinical practice guidelines recommend focused screening and expedited management of MDD in women (Guo *et al.* Obstet. Gynecol 2018; 131(4) 671-679). Despite this, drug labelling or dosing recommendations rarely account for gender or physiological differences between sexes, even though sex steroid level variations can impact drug absorption, distribution, metabolism, and activity both pharmacokinetically and pharmacodynamically (Soldin *et al.* Clin Pharmacokinet. 2009; 48(3): 143–157). Sertraline, an SSRI approved for the treatment of MDD, is one of the safer agents which can be given to childbearing or breastfeeding women (Cuomo *et al.* Expert Opin Drug Saf 2018; 17(7) 719-725). However, studies on the efficacy of sertraline for the treatment of MDD among women of childbearing age are limited.

**Objectives:**

This post-hoc pooled analysis evaluated the efficacy of sertraline in women with MDD, with a particular focus on women of reproductive age.

**Methods:**

A pooled data analysis of 8 short-term clinical studies of sertraline in persons with MDD (comprising 1600 participants from North America and Europe, of whom 947 were females; with moderate to severe MDD [mean±SD baseline HAM-D17 score was 23.73±3.58 for sertraline and 23.37± 3.47 for placebo]; sertraline dose, 50-200 mg) was performed. HAM-D17 total score was used to assess the efficacy of sertraline compared with placebo. The study period was 8 weeks. An MMRM method was used to analyse changes over time and ANCOVA to evaluate the change from baseline at week 8 with LOCF employed to manage missing data.

**Results:**

The analysis set consisted of 947 women (sertraline, 612; placebo, 335). The change from baseline in HAM-D17 total score was significantly higher for sertraline than for placebo at the end of 8 weeks (LS mean difference, 95% CI: -1.81 [-3.01, -0.62], p=0.0029, Figure 1A). This change from baseline was statistically significant starting from week 2 and increased over time (Week 2-8; Figure 2A).

The analysis set for women of child-bearing age consisted of 572 participants aged 18-44 (sertraline, 359; placebo, 213) from 7 clinical studies. The change from baseline in HAM-D17 total score was significantly higher for sertraline than for placebo at the end of 8 weeks (LS mean difference, 95% CI: -2.08 [-3.52, -0.64], p=0.0047, Figure 1B). This change from baseline was statistically significant starting from week 2 (Figure 2B).

**Image:**

**Figure:**
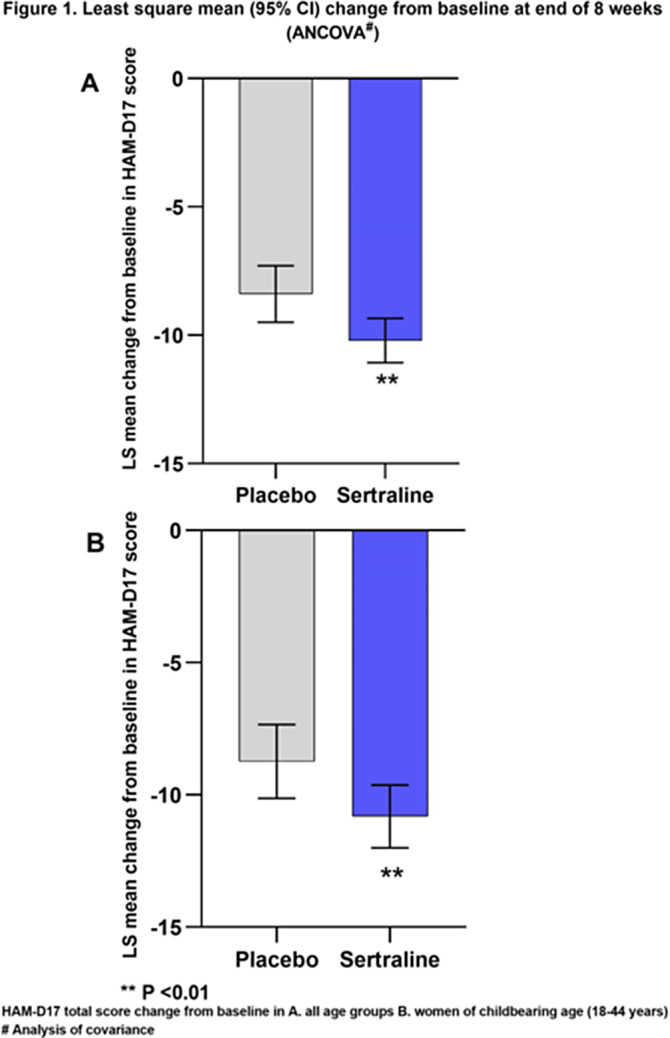

**Image 2:**

**Figure:**
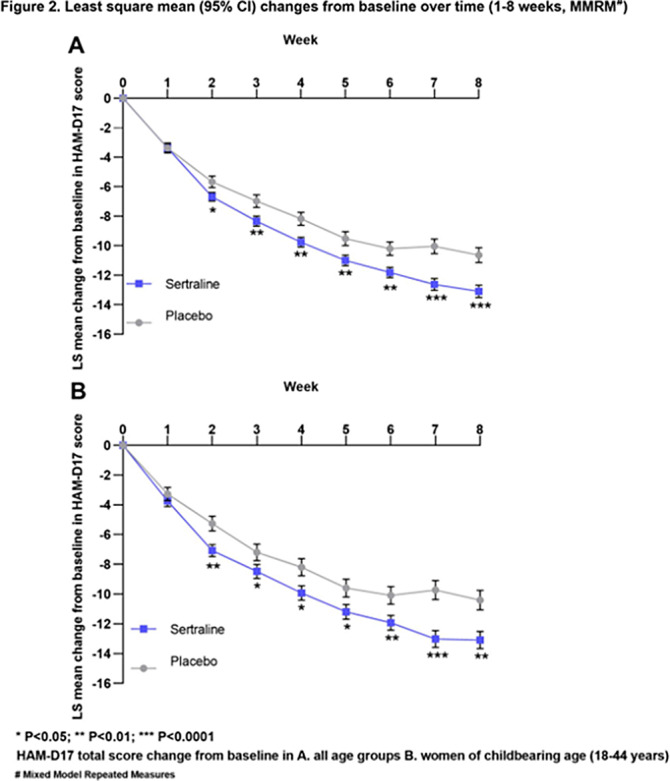

**Conclusions:**

Significant improvement in HAM-D17 scores was observed in the analysis, suggesting that sertraline is efficacious in treating women with MDD, including those in the childbearing age.

**Disclosure of Interest:**

A. Fagiolini Grant / Research support from: Angelini, Boheringer Ingelheim, Janssen, Consultant of: Angelini, Biogen, Boheringer Ingelheim, Lundbeck, Janssen, Mylan, Neuraxpharm, Otsuka, Pfizer, Recordati, Rovi, Sanofi Aventis, Viatris, Speakers bureau of: Angelini, Apsen, Biogen, Boheringer Ingelheim, Glaxo Smith Kline, Lundbeck, Janssen, Mylan, Neuraxpharm, Otsuka, Pfizer, Recordati, Rovi, Sanofi Aventis, Viatris, Vifor, M. Mariano Consultant of: Johnson & Johnson, Otsuka Pharmaceutical Inc., Speakers bureau of: Viatris Pharmaceuticals, Inc. Otsuka (Philippines) Pharmaceutical, Inc. H. Lundbeck A/S, Johnson & Johnson Philippines, Inc., E. Biesheuvel Employee of: Viatris Inc. Netherlands, P. Purushottamahanti Employee of: Viatris Inc. India

